# Fostering public health ethics awareness among medical students through interactive web-based values exchange learning: A cross-sectional study

**DOI:** 10.1097/MD.0000000000035808

**Published:** 2023-10-27

**Authors:** Tamara Kudaibergenova, Kenesh Dzhusupov, Nityanand Jain

**Affiliations:** a Department of Public Health and Healthcare, I.K. Akhunbaev Kyrgyz State Medical Academy, Bishkek, Kyrgyz Republic; b Department of Public Health, International Higher School of Medicine, Bishkek, Kyrgyz Republic; c Statistics Unit, Faculty of Medicine, Riga Stradinš University, Riga, Latvia.

**Keywords:** interactive learning, learning environment, medical students, qualitative research, research ethics, values exchange

## Abstract

Medical ethics have traditionally encountered resistance in medical education, with doubts regarding their necessity during preclinical years. Concerns include the practical application of theoretical ethics and favoring the learning of ethics through observation and imitation. Furthermore, ethics courses often focus on codes and regulations and neglect the promotion of moral reasoning and decision-making skills. Therefore, enhancing ethical researcher-training modules is essential for advancing instructional methods and promoting a unified and interactive learning atmosphere. A 3-week course was conducted using the values exchange (VX) online platform to assess the effectiveness of the VX system in involving students and its impact on their perceptions of the importance of research ethics. Accordingly, a blended course was provided as an optional course to the students, and a qualitative course evaluation survey was administered at the end of the course. Sixty-one medical students participated in a pilot-blended research ethics program and the majority (93%) reported a positive learning experience. The course was preferred over traditional classroom-based courses by 80% of the respondents, with 77% reporting increased interest and motivation to study Research Ethics. Over half of the students (57%) reported that the course stimulated their thinking and changed their perspectives on handling medical research issues. Some students (20%) reported improvements in their social and information technology skills. The VX platform was able to meet the expectations of both faculty and students, and fulfilled all dimensions of the Health Professions Learning Environment Conceptual Framework. The VX platform provides an interactive and effective simulated learning environment that promotes ethical research aligned with the Health Professions Learning Environment Framework and fosters core competencies, such as ethical reasoning, decision-making, and cultural respect. Medical schools are encouraged to implement VX to enhance the teaching of research ethics.

## 1. Introduction

Medical ethics (and bioethics) have traditionally been an unpopular subject among medical students and faculty, questioning the need for a dedicated, separate course in the medical curriculum.^[1,2]^ The effectiveness and impact of acquired theoretical ethical knowledge in practical situations has been among the cornerstone arguments against the inclusion of the course before clinical training.^[3]^ Students have argued that ethics are more naturally acquired through the observation of ethical behavior and imitation of role models.^[3–5]^ From a pedagogical standpoint, the faculty has repeatedly expressed doubts regarding the feasibility of teaching methodologies and the evaluation of acquired ethical knowledge.^[6]^

Furthermore, ethical debates can be challenging due to divergent priorities and values among societies, faiths, cultures, traditions, and organizations.^[7]^ At the same time, the relative effectiveness of research ethics courses can vary due to differences in the specific objectives, core knowledge, and skills expected of students.^[8–13]^ In addition, the information provided in ethics courses tends to focus on ethical codes and regulations,^[14]^ delivered through didactic lectures to large cohorts of students, which do not enhance an individual’s moral reasoning and ethical decision-making skills, the core competencies of an ethical physician-researcher.

Indeed, the education and training of future ethical researchers continues to be a demanding undertaking.^[15]^ A major reason for this is that repeated thinking, rethinking, and reflection are required. This is difficult to achieve in traditional classroom settings with a fixed length of time. As a result, research ethics courses fail to train ethical researchers, leading some researchers to practice a “check the box” exercise to get over the “hurdle” of ethics approval, increasing the risk of unethical research conduct and harm to research participants.^[16]^ The rapid adoption of science and technology, public involvement in shaping policies and state narratives, and increasing burden of chronic diseases have changed students perceptions of the importance of medical ethics.^[2,17,18]^ The coronavirus disease 2019 pandemic has accelerated this newfound interest and demand in the ethics and legalities of healthcare.^[2,19–21]^ Perhaps it reflects the shift in the focus of ethics in clinical practice from patient–centered care to greater-good utilitarianism.^[22,23]^

Some researchers believe that the implementation of effective instructional methods can enhance the quality and uptake of research ethics courses. A recent study identified the most and least effective instructional methods used in research ethics courses.^[9]^ However, the authors argue that these approaches may pose challenges because the effectiveness of a specific delivery method can be influenced by other training elements, such as the trainer’s expertise and instructional content.^[9]^ Considering these limitations, we believe that a research ethics course requires mastery of more than just separate, effective learning elements. Rather, an effective research ethics course requires a cohesive learning environment in which all elements are consistent, intertwined, and integrated.

As defined by Gruppen et al^[24]^, such an environment “refers to the social interactions, organizational culture and structures, and physical and virtual spaces that surround and shape the learners’ experiences, perceptions, and learning”.^[25]^ Accordingly, we piloted the values exchange (VX) tool, a unique web-based tool for fostering social debate,^[26]^ at our institution and found it to be valuable in achieving the above-described interactive learning environment. Hence, in this study, our objective was to assess the effectiveness of the VX system as a positive learning environment for students seeking to become ethical physician researchers. Furthermore, we aimed to identify the features of the VX system that could cause changes in student behavior.

## 2. Materials and methods

The study protocol was approved by the Research Ethics Board of the International Higher School of Medicine (approval no. 8, dated 02.11.2011). Informed consent was obtained from all the participants.

### 2.1. Study participants

This study was conducted at the International Higher School of Medicine (IHSM) in the Kyrgyz Republic, which offers a 5-year graduate program in medicine. The IHSM had a total of 4260 students enrolled in fall 2022, with about 400 students per academic semester. Since the school offers Public Health and Ethics as a compulsory course for all 4th-year (preclinical) medical students, we opted to use convenience sampling to recruit students from the semester. Accordingly, 61 students voluntarily completed the blended course and the course evaluation survey. Sample size calculations were not required in the present study because of the qualitative nature of the study design.

### 2.2. Research ethics course module

The teaching module consisted of 3 steps. The first step involved designing a blended 3-week course on VX Research Ethics to introduce medical students to research ethics and acquaint them with the virtual ecosystem of the VX system. In the second step, we conducted a course where Week 1 included classroom sessions with 2 didactic lectures: Introduction to Research Ethics and Ethical Decision-Making and Introduction to the VX system with practical exercises on the VX community website.^[26]^ Week 2 and 3 included e-training on the thematic VX assignment case - “Consent or the public interest?” (Table 1). This case was developed based on an ethical dilemma in public health research by Honorary Professor David Seedhouse (Aston University, United Kingdom), the creator and owner of the VX system.

**Table 1:** Description of the values exchange (VX) thematic assignment case.

CONSENT OR THE PUBLIC INTEREST? *Case description* Over the last 25 years, Human Immunodeficiency Virus/Acquired Immunodeficiency Syndrome (HIV/AIDS) has spread from a few widely scattered locations to become a truly global challenge. High rates of prevalence in certain countries—particularly those in India and Pakistan–mean that almost every business with local operations is affected in some shape or form via: • Their workforces—from junior employees to senior managers. • Their consumer base—through depopulation, decreased economic activity, and decreased disposable income. • The communities in which they operate—through contact with local people, contractors, and service providers. • Raising expectations that locally operating multinational companies (MNCs) should be acting against HIV/AIDS. A multinational drug company has approached the Indian government with a plan to cure HIV/AIDS. It says it has developed a prototype drug that it wants to put in the water supply of a select number of large factories to conduct a mass trial. The company says it is 90% sure this is a cure for HIV/AIDS, but it needs mass data. The drug company says the drug carries no risk but for such a large-scale trial informed consent is impossible. The drug company also wants access to the health records of all employees, in the interest of the trial and the public good. The company says this is the only way they can be sure the drug really works. Proposal: It is proposed that the Indian government should agree to the secret trial. Do you agree or disagree with the proposal? What should be done?

Students used the VX system to analyze a given case, reflecting individually, and collaborating within a group to enhance their understanding of the case. The group then shared their insights into the whole-class debate. The final step entailed collecting course evaluation survey responses from students upon completion of the course in Week 4. The questionnaire, administered in English, consisted of a mix of 18 closed- and open-ended questions covering demographics, overall opinion on the VX course, opinion on the VX system for learning Research Ethics, and a comparison of the VX course to traditional classroom-based courses.

### 2.3. Identification of thematic dimensions

To assess the efficacy of the VX system as an innovative method for imparting a Research Ethics course, we employed the Health Professions Learning Environment Conceptual Framework.^[24,25]^ The framework was selected because it is primarily designed for prospective evaluation and enhancement of a learning circuit. It identifies 5 core components that overlap and interact, forming 2 dimensions: psychosocial (personal, organizational, and social) and material (physical and virtual). Accordingly, we grouped the survey findings into their respective components of these 2 dimensions.

### 2.4. Data analyses

For closed-ended questions, we used a 5-point Likert scale to evaluate the learning engagement capacity of the VX platform (1 = little emphasis, 5 = great emphasis). The data from the Likert scales were summarized using the mode as the measure of central tendency. For the open-ended questions, we conducted directed content analysis (qualitative data). The open coding of data and quantitative text analysis were performed using MS Excel spreadsheets. We identified an “X” in each column corresponding to the categories mentioned in a particular response and added additional categories as the analysis progressed. Our unit of measurement was individual students and we calculated the percentage of responses that referenced each category. To identify the features of the VX system that could encourage learning among students, we used the VX platform. Furthermore, we linked learning effects and relevant VX system features to determine the optimal VX system-based learning environment for ethical research training.

### 2.5. Data validation for qualitative responses

The coding process for the qualitative data was evaluated by our colleagues to validate the accuracy of initial coding. A panel of outside observers, consisting of professors, statisticians, and students, peer reviewed the entire process. None of the observers was involved in the data collection process. Disagreements within the panel were resolved through discussion. The disagreement rate was < 3%. To enable generalization across various environments, we presented the study’s preliminary results to 6 nonrespondent 4th-year medical students and asked them to rate the similarity between the study’s results and their personal experiences.

## 3. Results

Our course and evaluation survey were completed by 61 undergraduates 4th-year medical students with a mean age of 23.17 years (range 20–29 years). More than 2/3rd of the respondents were male (69%). The responses to the questionnaire’s closed-ended questions indicated that the students had a favorable opinion of the VX Research Ethics Program, including its quality, organization, presentation approach, and appropriateness of the VX assignment case (Table 2). Participants found the VX platform to be an effective tool for learning about Research Ethics. The platform stimulated critical thinking and prompted questioning while also increasing interest and motivation to study Research Ethics. Overall, the course improved participants knowledge of Research Ethics and changed their understanding of certain medical research quandaries.

**Table 2:** Summary of the responses pertaining to psychosocial dimension from the Likert scale questions (*respondents* n* *= 61).

Thematic Component	Statement	Likert scale (No. of responses)						Dimensional component*	Dimension sub components*
		Strongly disagree	Somewhat disagree	Neutral	Somewhat agree	Strongly agree	% Strongly agree		
VX Platform	Very effective for teaching Research Ethics	0	1	4	14	42	69%	Personal	1
	Increased my interest and motivation to study Research Ethics	0	0	0	17	47	77%	Personal and social	1,2,5
	Stimulates thinking and encourages questioning	1	5	4	16	35	57%	Personal and social	1,5
VX Research Ethics Course	My opinion of the course after completion is positive	0	0	0	4	57	93%	Personal and organizational	1,4
	Overall, the quality of the course was excellent	0	1	1	10	49	80%	Organizational	4
	Considering the nature of the course and the method of presentation, the course was well organized	0	2	3	18	38	62%	Organizational and social	4,5
	The syllabi clearly laid out the expectations and contents of the course	0	1	4	21	35	57%	Organizational	4
	The VX assignment case was reasonable and appropriate in terms of content	0	1	2	10	48	79%	Personal and organizational	2,4
VX Course Effectiveness	My knowledge in this area increased because of this course	0	1	1	15	44	72%	Personal	2
	I changed my mind concerning some medical research issues after the course	2	4	4	16	35	57%	Personal and social	1,2,3,5

1 = Personal growth and goal direction, 2 = Identity formation, resilience and well-being, 3 = Engagement and emerging autonomy, 4 = Organizational, 5 = Social, VX = values exchange.

* The dimensions are based on the health professions learning environment conceptual framework.

### 3.1. Thematic dimensions

Our findings revealed that the VX platform and assignment case fully supported and achieved all dimensions and components of the Health Professions Learning Environment Conceptual Framework (see Supplemental Digital Content, http://links.lww.com/MD/K475). The VX learning environment was able to fulfill all the components of effective learning in the psychosocial and material dimensions. Open-ended questions revealed that 36% of the participants were deeply engaged in the course, 15% showed enthusiasm and eagerness, and 20% reported improved social skills. The course was favored over traditional classroom-based courses by 80% of the respondents due to its provision of new information, affording more time for reflection and responses, its global reach and advanced content, as well as serving as a superior platform for self-expression. Additionally, the students reported that the course was accessible and provided a wealth of knowledge, proving far more engaging than their traditional counterparts.

Regarding the material dimension, the physical space components of the IHSM building included classrooms, lecture halls, computerized testing rooms, and IT technical support, all of which are adequate for learning and practice. Additionally, the virtual space component, which pertains to the adequacy of virtual space for online learning, included provision for a browser, internet connection, and computer networks. The VX platform offers all electronic learning environments, digital assistants, and curriculum management tools necessary for interactive learning. This platform also includes mobile applications.

**Figure 1.**
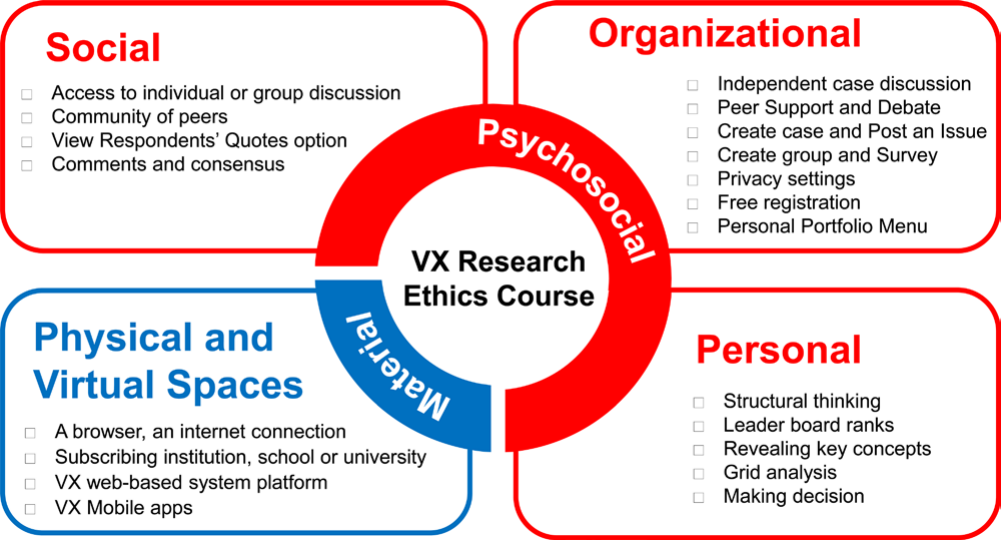
The conceptual model of the values exchange health professions learning environment for research ethics education. The concluded learning environment consists of two dimensions (psychosocial and material) and five components (personal, social, organizational, physical space and virtual space).

Based on these findings, we developed a conceptual working model of the VX learning environment that enables interactive learning when teaching medical ethics to future physician researchers (Fig. 1). We believe that the course was successful because it used practical teaching techniques, including engaging everyone by active participation, a blend of solo and team assignments, case studies, and less dependence on lectures.^[9]^ This was distinct from traditional teaching methods, which are generally too theoretical and mainly transfer knowledge from teachers to students (1-directional conversation; monologue) without encouraging their capability to make moral judgments in research. The VX system taught students to think ethically, make ethical decisions, care for other people’s values, learn about Research Ethics, and be aware of national and international regulations in an interactive simulated environment.

## 4. Discussion

In this study, we determined that the VX online platform is a useful and constructive learning environment for teaching Research Ethics to medical students. The results of the present study are unique. While previous studies have exposed health professionals in nursing, radiology, pharmacy, and social care to the VX platform and determined it to be an effective tool for navigating ethical dilemmas and decision-making,^[27–32]^ none have investigated its potential as an educational tool for promoting ethical research practices in public health among medical students.

Our results revealed that the VX platform successfully captured students’ attention and facilitated their comprehension of ethics in research. The underlying technology sparked inspiration among students, who recommended its implementation in other institutions globally to promote participation, discussion, socialization, and the development of ideas. Students found the VX platform to be stimulating and pertinent to value-centric decision-making. The software facilitated analytical thinking and encouraged the sharing of ideas and opinions regarding diverse social issues. This raised awareness of personal values, improved debate skills, and guided the justification of opinions while considering alternative perspectives from other participants.

Students preferred the web-based system over traditional classroom-based interactions because of their robustness and accessibility. Additionally, it allowed them to respond in their own time and gave them access to responses from others in a noncompetitive environment. The graphic user interface was both attractive and efficient, promoting clear thinking and the rational presentation of ideas. Students reported that the only benefit of traditional classroom-based courses that could resist VX implementation was the teacher’s ability to provide detailed explanations. Furthermore, some students desired a correct or recommended response for ethical cases, but this assumes a “*right answer*,” which is rarely the case in ethical reflection. Some also mentioned that the VX platform could be time-consuming and somewhat complex, but this could potentially merely reflect the nature of ethics themselves rather than a particular issue with the system interface.

Based on our experience, we believe that the VX platform inherently provides mechanisms to promote a positive interactive learning environment. It offers psychosocial support to learners through access to individual or group discussions as well as privacy settings during registration. The Personal Portfolio Menu includes options such as Profile and Portfolio, News Feed, Boards, My Friends, Messages, Groups, Peer Support and Debate, and Post Issue. Every saved case is automatically added to the user’s personal profile favorite. Students gained access to a detailed description of an issue with a suggested discussion proposal from the case author. The “*View Respondents’ Quotes*” feature offers the chance to communicate with anyone involved in the case. Respondents have the option to “*Like*” and “*Comment*,” imitating the social media communication interfaces that the students are used to. These VX system features promote resilience because individuals who can show empathy, inspire confidence, and build trust in others are generally more resilient.^[33]^

Another unique feature of the VX platform is its “*Grid Analyses*” tool, which utilizes colorful screens such as “*Basics*,” “*Reactions*,” and “*Emotions*” to enable users to organize their thoughts and identify key concepts, comments, and points of consensus when making decisions and solving problems. The VX platform is the only software that provides this tool and has the potential to foster ethical virtues among students. This characteristic is indeed quite impactful, as it suggests that machine learning can have a positive impact on human character development. Nonetheless, Aristotle posits that virtue develops through constant exercise. Hence, we propose the creation of a virtual simulator. Our intention was to provide a virtue trainer for students and professionals alike, especially in this era of morally disruptive technologies. To counteract the increasing appeal of the pharmaceutical industry and the risks of harm from unethical clinical trials, such trainers are imperative. To manage genetic technologies capable of affecting the biological nature of humans and avoid the risk of harm to the human gene pool, it is crucial to prevent the unethical use of artificial intelligence and deep-learning tools in science, including the writing of dissertations, development of weapons of mass destruction, and manipulation of human will.

Finally, the VX platform’s “*Leaderboard*” function encourages personal development among students. It works by ranking students activities based on collected points and granting honorary titles such as “*sage*,” “*genius*,” and “*boss*.” Students can view all user rankings based on their achievements. The VX platform contributes to the development of students professional identities by offering essential elements of meaningful learning.^[8]^

(i) **Activity**: Working on the case in the VX platform is a cognitive process in which the student plays a central role. Students actively engage by interacting with the case content and participating in subject matter discussions, making a personal cognitive contribution.
(ii) **Constructiveness**: The VX platform constructively provides students with the opportunity to construct their own meaning continuously by interpreting and reflecting on observed phenomena, case content, and the outcomes of their actions.
(iii) **Intentionality and Independence**: Students are encouraged to take individual ownership and initiative, be self-directed, consciously set goals, and commit emotionally without subjective evaluations.
(iv) **Authenticity**: The VX platform provides authentic or simulated experiences in a realistic context, ensuring that experiences become personalized and transferable.
(v) **Collaboration**: The VX platform facilitates social interactions between students and teachers, allowing for group collaboration and peer discussion within knowledge-building communities. The emotional involvement of students was also positively influenced by passionate and engaged teachers.

These features make the VX platform ideal for problem-based and case-based learning, as well as competence-based medical education. Another related ecosystem is the “*VX community*,” which represents a group of peers and individuals with a shared interest in the VX platform. Apart from the learning elements of students, student-to-faculty/staff elements include trust, feedback, clear expectations, communication, instructional strategies, and mentoring. Furthermore, there are elements between the students and patients that involve responsibility, acceptance, and trust. The quality of these interactions is characterized by social components, including equity, inclusion, and the problem of harassment and abuse by learners. All these social relationships influence what and how students learn, shaping their perceptions and experiences within the learning environment.

The VX platform fostered trust in a regulated learning environment. The “*Privacy Settings*” feature offers the option to use either a real name or pseudonym. Student-teacher communication is unbiased, with teachers able to discuss a case with students, but their responses were only revealed once students completed their own. The platform features a “*Peer Support and Debate*” function that allows students to comment and ask their teachers questions. The “*Post an Issue*” feature enables students to create their own issues or cases for discussion, providing support for discussions on uncertainties and obtaining the opinions of their peers and teachers both locally and worldwide. This feature aids in problem-solving and coping with uncertainty.

Our research reinforces these findings regarding the advantages of computer-supported collaborative learning. Effective learning results from the fusion of technological, pedagogical, and social factors within a virtual learning environment.^[34,35]^ Positive attitudes toward technological learning methods have been shown to increase students perceived enjoyment of using various tools for learning purposes.^[36–38]^ Proper planning and implementation of computer-supported collaborative learning promotes student satisfaction, influencing not only cognitive but also emotional aspects of the learning experience and its outcomes.^[39,40]^ The perceived simplicity of implementing computer-supported collaborative learning, as well as perceived benefits, had a positive impact on attitudes and perceived enjoyment. Both educators and students must understand these aspects to achieve success.^[38]^

On the VX platform, students are expected to review the case independently and then work together to merge individual perceptions, leading to a better understanding of the case through facilitated group conversations, and ultimately presenting their observations in a class debate. As such, the learning outcomes, in addition to the subject knowledge developed by the student from the case, include the acquisition of generic skills, such as communication, critical thinking, creativity, self-directed learning, collaborative or group learning, literacy in information technology, and other resources, and the development of higher-order thinking skills. Contemporary and realistic case studies provide students with the opportunity to witness theory in practice, adding meaning to their learning experience. These effects promote and encourage ethical behavior, cultivating ethical researchers.

### 4.1. Practical implications

In our teaching module, medical students had the opportunity to develop their skills as researchers of ethical physicians through collaborative learning and group discussions. They were trained to take responsibility for and respect diverse values and viewpoints. Medical schools can consider implementing the VX learning environment in their curriculum to teach research ethics as well as other allied specialties, including dentistry, physiotherapy, nursing, and pharmacy. This can be done in both the preclinical and clinical phases of education.

The VX learning environment is based on active teaching methods that prioritize student-centeredness. It offers easy access to teachers and peer support, which is instrumental in preventing exhaustion-related burnout and mental health issues commonly experienced by medical students and faculty members.^[41,42]^ An objective and reasoned understanding of students grasp of the concepts can be maintained by excluding subjective evaluations. In addition, the VX platform displays the responses of peers in the assignment case, revealing their values and decision-making processes. This characteristic can foster a growth mind-set among students, enhancing their involvement in the learning process and academic performance across all other medical majors. Adopting a growth mind-set could contribute to fostering lifelong adaptive behavior among future healthcare professionals.^[43,44]^

Although we demonstrated the effect of the VX platform on public health research ethics, it is also applicable to any field of education and research, as shown in previous papers describing ethics in radiology.^[31,32]^ It remains to be determined whether integrating strategic components of the learning environment that specifically address ethical uncertainty during undergraduate training would effectively prepare students for the pervasive uncertainty present in clinical practice.^[45]^ Nonetheless, anticipation and preparation for uncertainty in clinical practice can enhance students self-efficacy when facing such unpredictability and promote their overall well-being.^[46,47]^

### 4.2. Limitations and future directions

Our study has several limitations that are worth noting. First, our data only reflect the experiences of students at a single urban medical school, which may affect the generalizability of our findings across different educational systems. Additionally, the small sample size and use of convenient sampling also restrict our ability to generalize the findings. Gender, education level, or study year differences may yield intriguing findings; however, we chose to adopt a more modest approach and benefit from the collective first-user experience. Further research would be beneficial to expand this study to additional medical schools for a more precise subgroup analysis and to establish how these connections manifest in larger cohorts of medical students.

In addition to expanding the implementation of the VX platform across Kyrgyz medical schools, our future goal is to utilize the platform and assess its potential to aid students in managing uncertainty during clinical practice. As students can independently create clinical cases on the VX platform, it can aid in evaluating and comprehending peer opinions and discussions. The VX learning environment can also be utilized by medical professionals for case-based learning when multiple approaches to handling a clinical case are possible with varying case outcomes or if a specific diagnosis has not been established.

The VX learning platform may assist students in tackling intricate issues, ultimately fortifying their adaptability and flexibility in acquiring new knowledge while also training them to devise novel solutions in emerging contexts. It is worth exploring the VX learning environment to improve student well-being. The VX learning environment allows for in-depth discussions of the medical school experience and serves as a platform for promoting health and wellness. Given that the concept of self-efficacy appears to be correlated with the capacity to endure ambiguity, any initiative to improve student well-being should include deliberate exposure, practical training, debriefing sessions, and role modeling to acquaint students with uncertainty.

## 5. Conclusion

Traditional pedagogical techniques in Research Ethics focus primarily on imparting knowledge through monologue lectures. They rarely allow students to place their knowledge in a social context or demonstrate how values are integral to evidence-based medicine. Our findings revealed that the VX platform is a highly effective, interactive learning environment that promotes ethical research values in a simulated setting. The platform supported all the components of the Health Professions Learning Environment Conceptual Framework. VX’s collaborative learning environment and group discussions enabled medical students to develop skills in responsibility, respect cultural and ethical relativism, ethical research, moral reasoning, and ethical decision-making. Medical schools should consider implementing a VX learning environment in their courses to facilitate effective education on Research Ethics.

## Acknowledgements

We thank the Union Graduate College (UGC)’s Advanced Certificate Program in Research Ethics for Central and Eastern European Countries for the introduction to the Values Exchange (VX) platform and support of this study. We would like to thank Emeritus Professor Martin Strosberg (UGC, New York, USA) for project mentorship, support, and advice on study design and data collection. We would also like to thank Honorary Professor David Seedhouse (Aston University, UK), the creator of the VX system, and its administrators, for the opportunity to use the platform in our study. Last but not the least, we thank Professor Vincent O’Brien (Manchester Metropolitan University, UK) for his support and advice on study design and data collection. Authors declare no conflicts of interest. The present study didn’t receive any funding.

## Author contributions

**Conceptualization:** Tamara A. Kudaibergenova, Kenesh Dzhusupov.

**Data curation:** Tamara A. Kudaibergenova, Kenesh Dzhusupov.

**Formal analysis:** Tamara A. Kudaibergenova, Kenesh Dzhusupov, Nityanand Jain.

**Funding acquisition:** Tamara A. Kudaibergenova.

**Investigation:** Tamara A. Kudaibergenova, Kenesh Dzhusupov, Nityanand Jain.

**Methodology:** Tamara A. Kudaibergenova, Kenesh Dzhusupov.

**Project administration:** Tamara A. Kudaibergenova.

**Resources:** Tamara A. Kudaibergenova.

**Software:** Tamara A. Kudaibergenova, Kenesh Dzhusupov, Nityanand Jain.

**Supervision:** Tamara A. Kudaibergenova.

**Validation:** Tamara A. Kudaibergenova, Kenesh Dzhusupov, Nityanand Jain.

**Visualization:** Nityanand Jain.

**Writing – original draft:** Tamara A. Kudaibergenova, Nityanand Jain.

**Writing – review & editing:** Tamara A. Kudaibergenova, Nityanand Jain.

## Supplementary Material


