# Supplementary material for: Fostering public health ethics awareness among medical students through interactive web-based values exchange learning: A cross-sectional study
Source: Medicine (Baltimore). 2023 Oct 27;102(43):e35808. doi: 10.1097/MD.0000000000035808 (PMC10615508; doi:10.1097/MD.0000000000035808)
Supplement: Supplementary file 1 [file medi-102-e35808-s001.docx]

**Summarized Feedback -** The course is good for “*learning*”; “*getting clear with the subject*”; “*thinking precise and helping to come to the point, exactly what we think*”*;* “*extremely relevant to the world issues we live in at this time*”; “*robustness*” and “*simple options*”;” way *of expressing the ideas in the form of options*”.

**Supplementary Table 1.** The Values Exchange Research Ethics Learning Environment Framework*.

| **Psychosocial Component** | | **Student Comments** | **The VX platform relevant feature description** |
| --- | --- | --- | --- |
| Personal | 1. Personal growth and goal direction | *“Doing more projects like these with more experienced doctors and people can help us to boost up our ethical values.”*  *“Gathering more information from other places from other universities.”*  *“We should be engaged more programmes of that kind.”*  *“More detail to say discuss ethics world.”*  *“My suggestion for improvement is that it should be introduced worldwide so that more and more people get involved in it so new ideas will come and it will help also.”*  *“The Values Exchange is being a good tool for us. In fact, it is making our thinking precise and helping us to come to the point, exactly what we think.”*  “*Yeah, I agree with you its not only stimulates our think and encourages us but also gives us motivation for gaining some thing out of it and improves our skills of debating on serious issues.*” | Leader board ranks:  Leader board feature ranks user activity according to the collected points by exercising cases and getting honor titles (sage, genius, boss, senior worker etc.). Leader board demonstrates all users’ ranks.  Structural thinking and making decision:  User select a case and presses the button “Respond”. Than they got to 3 think pages “Basics”, “Reactions” and “Reasons” consequentially. In the Basics screen: a user state whether they agree with the proposal and who matters most. In the Reactions screen a user is suggested to select wedges with ideas that help them say what they really want to and then define it. In the Reason screen a user is suggested to select at least one tile in the grid of reasons and then to define it. After filling these pages, a user presses the button “Submit” and gets an opportunity to write an alternative way to deal with an issue or better proposal. As soon as they submitted their response, they get on the page with common reports “Thank you for your ideas, now find out what other people think…” Here user can read everyone’s comments and reply to them; find out how people voted and their demographics; find out how people used reactions chart to express their feelings; to find out how people used the tiles to strengthen their thinking; find out who is most important to people; read people’s extra ideas and comments on them. |
| Personal | 1. Identity formation, resilience, wellbeing. | *“Values exchange technology is an excellent way of expressing the ideas and resolving the questions involving various fields of public health .....Public health study has a great ability to open the minds of the individual and think widely...Keep up the good work..”*  *“Yes, I agree with their ability for stimulating our thinking on the serious issue, which is indeed very helpful for the prospects.”*  “*It raises every one interest and motivates to study research ethics.*”  *“This course is amazing gives a nice touch to our professional life.”*  *“Good, I changed a lot concerning some medical research issues.....and everyone has an opportunity to express them selves....and good to see people from different countries in dis group.”* | Create case feature: user selects a colourful picture with case, gets access to description of an issue with suggested by case author proposal. As long as a user press the “Respond” button they get new screens “Basics”, “Reactions”, “Emotions” that help users to structure their thinking, reveal key concepts, grid analysis, comments and consensus in decision and solving the problem.  Every case a user selects saved to their favourites in personal profile. |
| Personal | 1. Engagement and emerging autonomy | “*The best thing about this is that we come to know about the view of different people about such issues which have an impact worldwide and we get some information out of it.*”  *“Good, I changed a lot concerning some medical research issues.....and everyone has an opportunity to express them selves....and good to see people from different countries in dis group.”*  “*Yeah, I agree with you its not only stimulates our think and encourages us but also gives us motivation for gaining some thing out of it and improves our skills of debating on serious issues.*”  “*It really enlightens the views about our social value.”*  *“The information, not only being adequate to make a logical deduction on the issue but was also unbiased and presented all aspects of the issue at hand, thus raising many questions in the inquisitive mind.”*  *“Yes, I agree with their ability for stimulating our thinking on the serious issue, which is indeed very help full for the prospects.”*  “*The best thing in life is that we should share our ideas and views by sharing we come to know what the other people think about this particular issues.”*  “*No difficulty at all its was good, and to do so, it was the first time I was doing so, but it was great experience it was an easy way to express our thoughts and feeling about any serious issue.*” | As soon as students register on the platform, they immediately join the community of thousands of people interested in the exchange of values.  VX system feature “View Respondents’ Quotes” provides opportunity for communication with everyone discussed the case. Respondents can use the “Like” and “Comment” options.  The VX system features: free access to any group discussion.  Privacy Settings option at registration (choice to stay with real name or pseudonym);  Personal Portfolio Menu, including Profile and Portfolio;  News feed;  Boards;  My Friends (with opportunity to find friends and message);  Messages;  Groups (with opportunity to create a group);  Peer Support and Debate (with opportunity to ask question and post it setting filters);  Post an Issue (with opportunity to create an own issue or case for discussion). |
| Social | Social Networking and Communication | “*Yeah, I agree with you its not only stimulates our think and encourages us but also gives us motivation for gaining some thing out of it and improves our skills of debating on serious issues.*”  “*It really enlightens the views about our social value.”*  “*What I liked best about this course was that the issues raised were extremely relevant to the world we live in at this time.*”  “*it can connect n educate the people as well as aware.*”  “*The best thing about this is that we come to know about the view of different people about such issues which have an impact worldwide and we get some information out of it.*”  “*The best thing in life is that we should share our ideas and views by sharing we come to know what the other people think about this particular issues.”*  *“That is an interesting field; we can be learned by this course that how to respect the Human being and another living organism*...” | Free access to any individual or group discussion.  Groups feature (with opportunity to create a group);  Peer Support and Debate (with opportunity to ask question and post it, setting filters);  Post an Issue (with opportunity to create an individual issue or case for discussion).  User pressed the button “Respond” they got to 3 think pages “Basics”, “Reactions” and “Reasons” consequentially. In the Basics screen: a user state whether they agree with the proposal and who matters most. In the Reactions screen a user is suggested to select wedges with ideas that help them say what they really want to and then define it. In the Reason screen a user is suggested to select at least one tile in the grid of reasons and then to define it. After filling these pages, a user presses the button “Submit” and gets an opportunity to write an alternative way to deal with an issue or better proposal. As soon as they submitted their response, they get on the page with common reports “Thank you for your ideas, now find out what other people think…” Here user can read everyone’s comments and reply to them; find out how people voted and their demographics; find out how people used reactions chart to express their feelings; to find out how people used the tiles to strengthen their thinking; find out who is most important to people; read people’s extra ideas and comments on them. |
| Organizational | Organizational | *“The information, not only being adequate to make a logical deduction on the issue but was also unbiased and presented all aspects of the issue at hand, thus raising many questions in the inquisitive mind.”*  “*The methodology of the course was great, helping to the promotion of value exchange.”*  *“value exchange technology is an excellent way of expressing the ideas and resolving the questions involving various fields of public health .....Public health study has a great ability to open the minds of the individual and think widely...Keep up the good work..”*  “*The Graphic user interface and its robustness with the peaty simple options helping to get ourselves clear with the subject*.”  “*it was very effective for learning about Research Ethics*.”  “*What I liked best about this course was that the issues raised were extremely relevant to the world we live in at this time.*”  *“I liked the way of expressing the ideas in the form of options.”*  “*No difficulty at all its was good, and to do so, it was the first time I was doing so, but it was great experience it was an easy way to express our thoughts and feeling about any serious issue.*”  “*It's not that different means last time in class there was some informative discussion between us and our teacher it was nice yap somehow its good to have an online debate like that.”*  *“when coming to ethics, the traditional classroom-based courses are boring, and they force us to sit in the class the entire period. They also demand our attention during the class and are also a good and effective orator to make the field interesting. While coming to the computer-based system, it is robust, we can hook with it whenever we like. The graphic user interface is attractive and efficient to make the job done.”*  *“Not comparable as its very helpful to be taught by a teacher and the discussion b/w classmates.”*  *“I did not get bored here.”*  *“I think the online study is good as for as the knowledge is concerned, we can get the latest researches on the internet. but for clarification of concepts, we need a teacher that can be available in the classroom”*  *“The leisure of free time to respond and to present our ideas in an isolated, non-competitive environment is excellently conducive to free thinking and clear, rational presentation of ideas”.*  *“Good, I changed a lot concerning some medical research issues.....and everyone has an opportunity to express them selves....and good to see people from different countries in dis group.”*  *“In this form, the students pay attention whereas in class rooms it seems to be very boring”*  *“I have gained some have a good experience from this online course as compared to the traditional classes because from this we came to know about the worldwide ideas.”* | Free access to any individual or group discussion.  Privacy Settings option at registration (choice to stay with real name or pseudonym)  Personal Portfolio Menu, including Profile and Portfolio;  News feed;  Boards;  My Friends (with opportunity to find friends and message);  Messages;  Groups (with opportunity to create a group);  Create case feature: user selects a colourful picture with case, gets access to description of an issue with suggested by case author proposal.  The VX structure provide communication of students with teacher without any influence on the opinion of students by teacher. Teachers can discuss the same case with students, but their answers visible only after students completely responded to the case. Feature of VX “Peer Support and Debate” (with opportunity to ask question and post it setting filters) let the students comment and ask questions the teachers. “Post an Issue” feature (with opportunity to create an own issue or case for discussion) provide students with help to discuss their issues in case of their uncertainty and get opinions of their peers and teachers both locally and worldwide, that will help them in problem-solving and coping with uncertainty. |

* The study findings grouped under the Health Professions Learning Environment Conceptual Framework’s two dimensions: psychosocial and dimension (Gruppen L.D. et al. 2019). The psychosocial dimension components: personal, social, and organizational. The material dimension components: physical and virtual spaces.
